# Stakeholder perspectives on task shifting to prevent mother-to-child transmission of hepatitis B: A qualitative exploration of midwife- and nurse-led interventions in Vanuatu

**DOI:** 10.1371/journal.pgph.0006450

**Published:** 2026-07-08

**Authors:** Leila Bell, Stephen Bell, Aleesha Kalulu, Malietasi Bulu, Nicole Allard, Kali Ameara, Emily Deed, Zeshi Fisher, Margaret Hellard, Caroline S. E. Homer, Kaylene Kalmos, Sereana Natuman, Harriet Obed, Harriet Sam, Mark Stoové, Annie Taissets, Ben John Taura, Florita Toa, Caroline van Gemert

**Affiliations:** 1 Burnet Institute, Melbourne, Australia; 2 Monash University, Melbourne, Australia; 3 Burnet Institute, Port Vila, Vanuatu; 4 Department of Infectious Diseases, University of Melbourne, Melbourne, Australia; 5 WHO Collaborating Centre for Viral Hepatitis, The Doherty Institute, Melbourne, Australia; 6 London School of Hygiene and Tropical Medicine, London, United Kingdom; 7 Department of Infectious Diseases, The Alfred Hospital, Melbourne, Australia; 8 Doherty Institute and School of Population and Global Health, University of Melbourne, Melbourne, Australia; 9 Ministry of Health, Port Vila, Vanuatu; Johns Hopkins University Bloomberg School of Public Health, UNITED STATES OF AMERICA

## Abstract

In Vanuatu, the management of hepatitis B is limited to hospital settings whilst antenatal care is decentralised to primary care settings, overseen by nurses and midwives. Scale up of interventions during pregnancy to prevent mother-to-child transmission (PMTCT) of hepatitis B in Vanuatu therefore requires task shifting of care for hepatitis B. This study aims to understand stakeholder perspectives on task shifting hepatitis B PMTCT interventions to midwives or nurses in primary care to increase access to services and decrease risk of mother-to-child transmission of hepatitis B in Vanuatu. Twelve semi-structured interviews were conducted with policy makers and key stakeholders involved in hepatitis B care at national and provincial levels. Interviews were recorded, transcribed and then translated into English. A deductive thematic analysis was undertaken. Key findings focused on acceptability, feasibility and sustainability. The importance of hepatitis B PMTCT was recognised and the need to improve access and coverage through task shifting was supported. Stakeholders felt nurses and midwives were highly trusted by the community and existing antenatal care roles were seen as supporting acceptability for hepatitis B task shifting. Supportive policies, guidelines and networks were required to enable nurses and midwives to provide hepatitis B PMTCT, some of which already exist. Stakeholders highlight the need to develop an enabling environment for sustainability, including appropriate resourcing, policies to formalise task shifting, pathways for communication and referrals, and increased client awareness and health education. There was support for the expansion of midwife- and nurse-led interventions for PMTCT of hepatitis B. However, there is a need for training, capacity strengthening and strengthening of supply chains to ensure continuity of availability of test kits and medication. Strengthening of these health system-level components will improve PMTCT efforts and broader maternal and child health.

## Introduction

Hepatitis B infection is most commonly transmitted from mother-to-child during pregnancy or in early infancy when risk of developing chronic infection is highest [1,2]. There is no cure for hepatitis B infection, so prevention of mother-to-child transmission (PMTCT) through testing, treatment and vaccination is a priority [3]. The island nation of Vanuatu has an intermediate to high burden of chronic hepatitis B, with an estimated prevalence among women of reproductive age of around 5% and approximately 3% in children under five years of age, highlighting continued risk of transmission from mothers to children [4]. Global targets for the elimination of mother-to-child transmission of HIV, syphilis and hepatitis B include achieving hepatitis B surface antigen prevalence of 0.1% or less in children five-years and younger [5].

In Vanuatu, there are four levels of health services that provide care to over 300,000 people living on 65 inhabited islands, with 78% of the population residing in rural areas [6]. Primary health care is provided at three health facility levels: aid posts, staffed by village health workers; dispensaries, which may be staffed by a nurse or midwife and a nurse aide; and health centres, which may have a nurse, midwife, nurse practitioner and a nurse aide. Not every health facility is fully staffed per the Ministry of Health Role Delineation Policy, with a health facility readiness and service availability assessment indicating only 28% of primary health facilities, specifically dispensaries and health centres, met the requirements in the national Role Delineation Policy in terms of adequate staffing [7–11]. Based on 2018 data, the ratio of skilled health workers (i.e., doctors, registered nurses, midwives) was 15.6 per 10,000 population, which is below the national target of 38.16 [12]. In 2019, there were a total of 48 medical doctors in the country (1.6 per 10,000 population), and 426 nurses and midwives (14.0 per 10,000 population) [10]. There are few medical doctors based in public primary care facilities, with most doctors within the government system working at the six public hospitals located in each province. There are also a small number of private clinics that provide primary health care, predominantly in urban areas and usually for a higher fee than government services.

A national decentralisation effort attempts to move appropriate healthcare from over-burdened hospitals to primary care facilities but challenges remain, including ensuring sufficient human resourcing and some client preferences for seeking care at hospitals over primary health facilities [12]. While there is a need to increase the number of healthcare workers, having nurses and midwives assume greater responsibility for delivering certain types of care would enable clients to receive care in a community-based health facility, without the need to travel to a tertiary health facility which is aligned with this effort to decentralise health services [12]. Task shifting involves moving responsibility of certain tasks to other disciplines of health workers to increase efficient use of human resources, overcome the challenges of health worker shortages and achieve Universal Health Coverage [13]. In many countries, task shifting has been applied to service delivery and care for HIV and hepatitis C [14–18]. In some countries, management of hepatitis B is also moving away from specialists towards general practitioners or nurses [19,20].

The scope of practice for nurses and midwives in Vanuatu is guided by the Health Practitioners Act 1984 and Nurses Act 2000 and allows for ordering of laboratory tests and some prescriptions, such as routine antenatal medications, or ongoing prescriptions of some medications after initial prescription by a doctor [21]. Hepatitis B services include testing of pregnant women during antenatal care (ANC), universal infant vaccination with hepatitis B birth dose and three doses of pentavalent vaccine, and some treatment with tenofovir disoproxil fumarate (TDF) [22]. ANC is provided in both hospitals and primary health facilities but hepatitis B testing and treatment are centralised at hospitals. Outside of an ongoing field trial [23], few pregnant women receive prophylaxis treatment for PMTCT of hepatitis B. This is despite expanded 2024 WHO guidelines that provide conditional recommendations for universal prophylaxis treatment for hepatitis B surface antigen (HBsAg)-positive pregnant women where there is no availability of hepatitis B viral load or hepatitis B e antigen testing [3], which is generally the case in Vanuatu.

Midwives and nurses provide the majority of ANC services for pregnant women in Vanuatu, while high-risk women (those with identified pregnancy complications or existing health conditions) receive care from doctors [24]. Clients are therefore accustomed to midwife- and nurse-led care during pregnancy. For improved PMTCT of hepatitis B in Vanuatu, nurses and midwives at a primary level could provide testing and hepatitis B prophylaxis treatment, increasing access significantly. However, policy makers’ perspectives on task shifting PMTCT interventions for hepatitis B to midwives or nurses in primary care facilities is not known. In response to these knowledge gaps, this study aimed to understand perceptions of policy makers on the feasibility, acceptability and sustainability of integrating interventions for PMTCT of hepatitis B during pregnancy into ANC provided by nurses and midwives in Vanuatu.

## Methods

### Study design

A qualitative study was undertaken. The intervention (sometimes referred to as “model”) being discussed was integration of hepatitis B testing and prophylaxis treatment into routine ANC care provided by midwives or nurses in hospital- and community-based health facilities. This would involve task shifting care from physicians at hospitals to midwives and nurses at all levels of health facilities.

The study was informed by an ‘interpretive’ approach to research which emphasises that people make sense of social phenomena (i.e., models of service delivery) through experience and interaction with others, and that qualitative methods can enable deeper understanding through attention to people’s perceptions and experiences in particular local settings [25]. The Consolidated Criteria for Reporting Qualitative Research (COREQ) checklist guided the reporting for this study (see S1 File) [26].

### Participants, sampling and recruitment

Between 10 December 2024 and 12 February 2025, data collection was conducted in three provinces of Vanuatu: Shefa, Sanma and Tafea. These are the three most populated provinces, where the three largest urban areas in the country are located. Referral hospitals are located in Efate Island in Shefa province and Espiritu Santo Island in Sanma province. These provinces have been prioritised by the Ministry of Health for expansion of PMTCT services and are also involved in the Protektem Pikinini Blong Yu Trial (“Protect your Baby” Trial), a single-arm field trial looking at the effectiveness of universal antiviral prophylaxis treatment for pregnant women living with hepatitis B for PMTCT of hepatitis B [23]. The trial is in a pilot phase delivered through research nurses outside the normal health system. Recruitment had started only in Shefa by the time the interviews were concluded.

Policy-level stakeholders were interviewed — some involved in PPBY study design and some aware through professional networks — but none are implementing the model in routine care. Study participants included provincial and national stakeholders involved in health policy and decision making at national and provincial levels. No clinical nurses or midwives were included. Potential participants were identified purposively from study investigators’ professional networks as having key roles and privileged operational insights relevant to hepatitis B programmes and care and were contacted in-person or via telephone or email to be invited to participate in the study. Twelve stakeholders from national- and provincial-level health services, including representation from public health and curative services, were interviewed and none dropped out.

### Data collection

Semi-structured interviews were conducted, using an interview guide which was developed in English (SB, LB, AK) and translated into Bislama by bi-lingual team members (LB, AK) and piloted among interviewer networks (see S2 File for English version). The topic guide was informed by two implementation science models – the Practical, Robust Implementation and Sustainability Model (PRISM) [27,28], and the Reach, Effectiveness, Adoption, Implementation, Maintenance framework (RE-AIM) [27–29] to seek a comprehensive view on task shifting, particularly crucial for resource-constrained settings addressing health inequities. Our interpretive approach recognises that stakeholders’ perspectives on service delivery models are shaped by their experiences within Vanuatu’s health system context. PRISM and RE-AIM provided complementary frameworks to help collect and analyse stakeholders’ perspectives collected during interviews: (1) PRISM helped us understand contextual factors (organizational characteristics, infrastructure, external environment) shaping sustainability of decentralization and task shifting, and (2) RE-AIM dimensions (particularly adoption, implementation, maintenance) helped examine whether these changes could support sustainable PMTCT outcomes. Both the PRISM and RE-AIM frameworks were applied prospectively, using stakeholder perspectives to identify implementation determinants and maintenance preconditions prior to scale-up should the PPBY trial show effectiveness of the prophylaxis-for-all approach for PMTCT, enabling early identification of system-level factors likely to shape programme success or failure.

Interviews were conducted by trained social researchers (AK, LB) in English or Bislama as per participant preferences, in audio-private settings perceived as safe by participants, at a time convenient to them. Interviews lasted between 16 and 68 minutes, were audio-recorded, transcribed and translated into English (by MB, AK, LB) and deidentified in preparation for thematic analysis. Translation was reviewed by one additional person fluent in both English and Bislama (AK, LB, MB) to confirm accuracy and interviewer consistency.

### Data analysis

Transcripts were uploaded into NVivo X15 for deductive thematic analysis guided by PRISM and RE-AIM models [27–29]. Thematic analysis followed Braun and Clarke’s [30] six stage process: data familiarisation by reading through the transcripts and making notes; generating initial codes; searching for themes; reviewing themes; defining and naming themes; reporting the outputs. Analysis was conducted by one researcher (LB) and then discussed with additional researchers (CvG, AK, SB) for interpretation of data to ensure consistency and understanding of the interview context and alignment with conceptual models.

### Ethical considerations

Ethical approval was granted by the Vanuatu Health Research Ethics Committee (no reference number, letter dated 25 October 2024) and the Alfred Health Human Research Ethics Committee (552/24). Participants provided written informed consent. Data were stored on a secure server. To protect participant identity, all data were anonymised through use of codes which are reported in this paper. Given the small number of individuals involved in hepatitis B policy and program implementation in Vanuatu, detailed descriptions of participant roles and organisational positions have been intentionally limited to protect participant confidentiality and reduce the risk of identification. Additional information regarding the ethical, cultural, and scientific considerations specific to inclusivity in global research is included in the Supporting Information (S1 Checklist).

### Author reflexivity statement

The core research team comprised two ni-Vanuatu and three international researchers. AK (BPH) is a ni-Vanuatu woman who has over 5 years’ experience working in public health, including as a researcher in Vanuatu, focusing on infectious disease surveillance, vaccine safety surveillance and maternal and child health. MB (BComDev) is a ni-Vanuatu woman studying in Australia, with a strong focus on grassroots community engagement. LB (MPH) is an Australian woman with 10 years’ experience in global public health programme implementation and research, including over a year living and working in Vanuatu. CvG (PhD) is an Australian woman who lived and worked in Vanuatu for 5 years and has 20 years’ experience in public health research, with a primary research focus on developing and evaluation strategies for PMTCT in the Pacific. SB (PhD) is a British man who holds Australian citizenship and has over 20 years’ experience in participatory community-based research across diverse settings, including in the Pacific. This study was a component of LB’s doctoral research; CvG is on LB’s supervision team and is the principal investigator of the PPBY trial. Other co-authors include staff at the Ministry of Health Vanuatu and international public health researchers. We recognise that our personal views and experiences influence our interactions with our work, the world and each other. Together we are a multidisciplinary team of researchers and implementors working to improve strategies for PMTCT of hepatitis B and improve the health and wellbeing of people living with hepatitis B.

## Results

**Fig 1:**
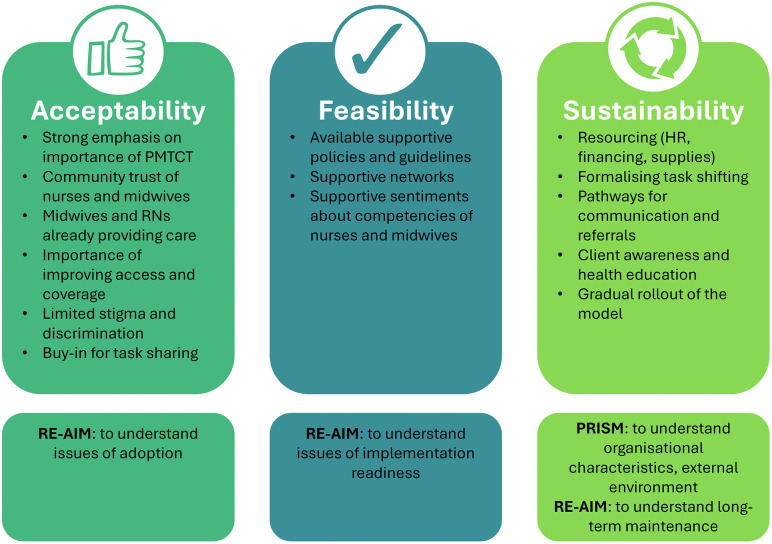
Stakeholder perceptions of acceptability, feasibility and sustainability of midwife- or nurse-delivered interventions to prevent mother-to-child transmission of hepatitis B during pregnancy in Vanuatu.

Stakeholders reported a range of factors influencing acceptability, feasibility and sustainability of midwife- and nurse-delivered interventions for hepatitis B PMTCT (see Fig 1).

### Acceptability

Task shifting to midwives and nurses was supported for a range of reasons. Stakeholders felt that PMTCT of hepatitis B was important and thought that mothers and providers understand why providing hepatitis B care to pregnant women through ANC is important. The prevention benefits are highlighted in the following:

“We talk about prevention is better than cure, and as we know hepatitis B is a viral infection that once we get it, we can treat the signs and symptoms, but we can’t cure it. […] Yes, if we can’t cure, at least we can cut this route of transmission to limit spread to the baby.” SH7

Stakeholders believed that community members trust nurses and midwives due to pre-existing care responsibilities. One stakeholder (SH8) explained, “they [the community] have full trust in our nurses in the health facilities, so they [the community] already know what services they provide, and they trust all of them.” Participants felt that task shifting care responsibilities from doctors to midwives or nurses for hepatitis B care during pregnancy would be well accepted by the community. As one stakeholder explained, community members do not tend to differentiate between the types of care or treatment they already received from doctors, midwives or nurses, saying:

“People in the community don’t know which treatment is for which disease. But they know that the nurses know the different treatments for the different diseases. So, I don’t think they would say the nurses are or are not allowed to do something, they put their full trust in the health officers in our health facilities.” SH8

Stakeholders explained how midwives and nurses working in health centres and dispensaries already have specific care responsibilities aligned with PMTCT, including routine ANC services, childhood vaccination and counselling of pregnant women to engage in care. As such, stakeholders felt that midwives and nurses are well placed to help decentralise the provision of care for PMTCT of hepatitis B, which would improve access and coverage of hepatitis B interventions and increase demand for testing and treatment. One stakeholder (SH11) said, “if we decentralise this service to the community, it will be very effective because a lot of women have challenges with the cost of travel to come to the hospital to attend their visits.”

Due to perceptions of limited stigma associated with hepatitis B, stakeholders felt that a community-based approach to accessing hepatitis B care, including for testing and treatment, would not be constrained by barriers associated with fear or gossip. One stakeholder explained that hepatitis B is “like a normal illness”, saying:

“If we treat it like something that is taboo, then that increases stigma and discrimination. But with hepatitis B, I think many people in the community will be happy because there are many cases in rural areas, we hear stories when we go. So, I don’t think there is much stigma and discrimination around hepatitis B.” SH2

In the national context of limited human resources, participants felt that doctors would appreciate extra support provided by nurses and midwives through this task shifting strategy. As an interviewee (SH2) explained, “If we decentralise and go to the clinics and health centres and give midwives the capacity to deliver this, it would be very helpful to the doctors. So, I think they would be happy.” There was enthusiasm among stakeholders who were excited about the possibility of midwife- and nurse-led PMTCT for hepatitis B across the country, with one participant (SH3) saying, “It’s a very good model, and we should hopefully be able to continue it.”

### Feasibility

Feasibility of shifting responsibility for PMCTC interventions for hepatitis B to nurses and midwives requires an enabling policy environment. The Vanuatu decentralisation policy, which aims to move care from overburdened hospitals to direct service provision in communities, could offer a first step to support task shifting. As one participant (SH6) explained, “Because our policy is that health should be accessible and affordable, we must try our best to have everyone be able to access services on their doorstep.” This would, however, require updates regarding roles and scope of practice, including around prescribing.

Stakeholders reported existing policies and guidelines, including the obstetrics guidelines and a flowchart for hepatitis B treatment, that provide a good foundation but would need to be adapted to reflect nurse- and midwife-led PMTCT for hepatitis B. A stakeholder (SH5) explained the need to, “review and update the guidelines. We can direct the midwives to it for proper management care.” An update of the policies and guidelines would need to be followed by an action plan and appropriate training to ensure adaptations to policies are implemented. It was also recognised that existing networks could facilitate uptake of this model and support task shifting. These included group chats and phone networks which could facilitate sharing of advice between nurses and midwives, as well as other senior members of the broader midwifery community and doctors. One participant (SH12) commented on how improvements in telecommunications nationally support community-level providers to be better connected to others. Another participant described existing support networks that could also be drawn on to support hepatitis B interventions, saying:

“When a mother is in labour for example, and they need advice they can put it in the group chat, and someone will respond. Everyone in the Vanuatu midwifery society is in the group chat. It is good to have a platform like this.” SH5

There are existing networks for nurses and midwives to contact doctors directly to seek advice, including about the care of pregnant women with complexities and possible referrals. One participant (SH1) said nurses “have the contact number of the clinicians, the doctors” available at their workplace, so if “they come across [a difficult case that requires clinical advice], then they just call and communicate with the doctors.”

Stakeholders felt that nurses and midwives were capable of providing different forms of care and learning new processes, as long as they are empowered and resourced to do so. One participant (SH11) said, “If we give them [the nurses] the information, if we empower them with the knowledge, and then give them the resources, they will be successful.”

### Sustainability

For long-term sustainability, participants highlighted challenges that would need to be addressed to feasibly implement decentralised, midwife- and nurse-led PMTCT hepatitis B interventions across Vanuatu. Understaffed facilities were highlighted as a systematic challenge facing the Vanuatu healthcare system. One participant (SH6) noted that “health centres should have midwives but sometimes don’t” and followed on by saying “sometimes a midwife is the only one who can do something, but some dispensaries don’t have midwives just nurses.” They explained that for the model to be sustainable, both nurses and midwives would need to be trained to overcome staffing gaps.

Resourcing challenges within facilities, including maintaining supply of consumables, was also perceived as a barrier to sustainability of decentralised care which would in turn increase access to and subsequently demand for services thereby requiring more resourcing. For example, one participant (SH11) said that in “many of the health facilities, the work environment for the nurses isn’t really safe for them to practice”, pointing to how the lack of consumables for infection, prevention and control measures may make it unsafe for midwives and nurses to conduct their duties. Other participants noted challenges with stock management and supply chains, particularly for facilities in remote and isolated areas. For example:

“Medication supply chain, we always have issues with med supply forecasting, yeah forecasting is one and actually getting this medication from CMS [Central Medical Store] to the provincial hospital, from provincial hospital down to the health centres.” SH3

Existing challenges with financing of health services were also highlighted as constraining an expansion of hepatitis B care. One participant (SH2) said, “I think the main challenge has to do with financing to implement this model. Finance is often a challenge for us.” Constrained budgets were perceived to limit the ability of nurses and midwives to provide individualised care to patients. One stakeholder explained:

“They [health workers] have their plans, like they might want to follow up some women who do not present [to care] anymore, and they live far away, but there is no budget for the nurse to plan an activity to go visit [the woman], this is a big challenge that the nurses are facing now. Especially for these small activities, to conduct outreach or make a home visit, budget plays an important role and it is a big challenge for them.” SH11

Additional concerns were raised about the need for continuing professional development for nurses and midwives in the community, to ensure they are practising best evidence-based care. One stakeholder (SH12) said, “things have changed. Up to date, evidence-based practice, they have no idea, they are doing their normal patient consultations.” Stakeholders highlighted how continuing professional development could be updated through job descriptions, standard operating procedures and ultimately integration of these expanded responsibilities into the nursing and midwifery curricula. This long-term possibility was described by one participant who said:

“In the long-term view, in terms of policy, we can include this in the nursing school curriculum. To make sure this training is captured during nursing school practicals, so that they know how to test and provide treatment. And during that time, they learn a lot, and then when they go to the field, we can just continue to provide them with refresher trainings just to upgrade their skills.” SH7

While there were some existing networks that workers accessed, participants described a need to strengthen support networks for confidential communication with doctors and to coordinate referrals of complex cases. For example,

“I think it is good for them to have support from the doctors at this stage when they come across a case with hepatitis and complicated conditions. I think it is good to maintain a support group for them with the doctors, to communicate and be able to refer the patients.” SH2

Stakeholders highlighted the need for additional efforts to encourage participation and adherence in interventions by increasing awareness and health education which would ultimately increase demand by clients. For example, one participant (SH5) explained the need to provide hepatitis B prevention information, education and communication to pregnant mothers so that they become “aware if they have a condition and they may be required to attend appointments, not just for the baby, but for themselves.” Another participant described how efforts would be required to enhance community-wide health literacy to engage all women in care:

“When a mother has a good educational background, when you explain something to her, she is able to understand. Other times, we take longer to explain to mothers because they have very little education, and even then, they still don’t understand why it’s important. So, they turn to kastom [traditional] medicine instead.” SH10

Sustainability depends on the gradual integration of hepatitis B testing and prophylaxis treatment into standard antenatal care. As one participant explained, there is an opportunity to evaluate and adapt implementation based on province- and health centre-specific realities on the ground:

“it is good to pilot first at a specific health facility before we start to use it, or we are in a position where everyone must provide this service. And then after you must evaluate, and we find the gaps and work to address then.” SH9

## Discussion

This study addresses a major gap in knowledge about task shifting in public health service provision in Vanuatu, exploring the acceptability, feasibility and sustainability of midwife- and nurse-led hepatitis B care during pregnancy. Our analyses illustrated strong stakeholder acceptability and perceived feasibility for this model of hepatitis B care. Acceptability was driven by strong recognition of importance of hepatitis B PMTCT and of improving access and coverage; community trust of nurses and midwives; midwives and nurses already providing the majority of ANC care; limited hepatitis B-related stigma and discrimination; and buy-in for task sharing across the clinical workforce. Perceived feasibility of proposed changes was driven by existing supportive policies and guidelines; supportive networks for seeking clinical advice; and perceived competencies of nurses and midwives. However, participants raised concerns about long-term sustainability of the model, related to effective resourcing to cope with expanded service provision and demand (HR, financing, supplies); introducing policies for task shifting; strengthening pathways for communication and referrals; enhancing client awareness and health education; and gradual rollout of the model.

Two implementation science frameworks were applied prospectively to analyse stakeholder perspectives on task shifting. PRISM was used to help understand the contextual factors shaping sustainability of decentralisation. RE-AIM was used to help examine whether adoption, implementation and long-term maintenance of midwife- and nurse-led PMTCT could be achieved and sustained. Interpreted through these frameworks, acceptability and feasibility findings reflect RE-AIM conditions for adoption and implementation. Sustainability findings, interpreted through RE-AIM, provide concern for long-term maintenance of the model. These findings, further interpreted through PRISM, reveal gaps in organisational characteristics, infrastructure and external environment that constitute implementation preconditions rather than simply future programme challenges. These system-level gaps raise an important question about whether Vanuatu’s health system is sufficiently ready for task-shifting at scale. This is consistent with experiences in other under-resourced and island settings where similar factors limited task-shifting efforts and successful task-shifting relies on also addressing the underlying systems issues [31–34]. Participants reported community trust in nurses and midwives which appears to support adoption. However, the noted gap in evidence-based practice at facility level signals a challenge of ensuring consistent, evidence-based practice across facilities, requiring active supervision and ongoing support structures, not just initial training or reliance on nursing college curricula. Together, these findings suggest that stakeholder support exists for this model, but health system strengthening is a precondition for sustainable scale-up, not an afterthought.

Task-shifting from doctors to nurses and midwives for a variety of health services has been found previously to be acceptable and feasible, including in resource-limited settings [35–40]. These findings add to the evidence base about potential task-shifting in island nations and where health care human resourcing is a challenge. Our findings on acceptability align with other research in Vanuatu which points to people’s trust in nurses as trained and knowledgeable sources on public health issues [41,42]. Our findings also align with sustainability challenges that have been documented in Vanuatu and elsewhere, including concerns related to limited human resources, challenges with rollout of training, and existing workload of nurse and midwives [15,21,43]; financial restrictions on sustained financing of midwife-led care models [15]; and challenges of supply chain management [21,44]. Thinking about long-term sustainability of this model, the human resource shortages need to be addressed, to avoid adding additional responsibilities to already overburdened staff and negatively impacting the provision of other essential services.

Contrary to our findings, other settings have found that stigma and discrimination is common and has a negative impact on people living with hepatitis B but this is not well described beyond communities in Asia or among immigrant populations [45,46]. The feasibility and advantages of midwife- or nurse-led care for hepatitis B have been demonstrated in other settings. For example, a retrospective cohort study of pregnant women living with high hepatitis B viral loads attending public hospitals in Hong Kong, found strong engagement from pregnant women in a collaborative care approach between obstetricians, hepatologists and hepatitis nurses. With nurses leading education around PMTCT, TDF uptake increased during pregnancy in this collaborative model compared to women receiving routine care [20]. While this model may not be feasible in Vanuatu because of the limited availability of these specialist physicians, our results have identified an alternative model that was considered feasible by major stakeholders which could contribute towards progress against hepatitis B elimination goals. Additionally, a recent systematic review and meta-analysis mostly from high-income countries, found that nurse involvement across 13 roles, broadly categorised as health education, case management, and nurse-led specialist clinics, achieved improvements in hepatitis B screening and detection rates, hepatitis B vaccinations, immunity rates and patient adherence with treatment and monitoring [47]. Our findings highlight the potential for similar improved hepatitis B care outcomes through increased involvement of nurses and midwives especially at a community-based level.

This study highlights the need for further health systems strengthening before any task-shifting of PMTCT interventions for hepatitis B. The Vanuatu Ministry of Health role delineation policy [7] designates that health centres should have both a midwife and a nurse, but over 70% of facilities have vacant positions [11]. Any expansion of hepatitis B care, therefore, must consider human resources, as well as the additional costs for procurement of test kits and TDF, as well as additional costs for shipment, referral, and advance hepatitis B-associated care. However, while costs may increase driven by enhanced access and demand, the cost per episode of care will be cheaper than centralised doctor-led models. Given that findings from our interviews identified many of the same challenges of supply chain management that were identified 15 years ago [21,44], there are existing broader system-level challenges that continue to require strengthening. Additionally, natural disasters in Vanuatu are common, and while a decentralised approach will be more resilient to these impacts, there remains a need for preparedness efforts to reduce the impact on supply chains and clients’ access to healthcare. Should the prophylaxis-for-all approach for hepatitis B PMTCT be found to be effective, policy makers could consider a stepped approach to facilities that have sufficient human resources, including to conduct hepatitis B testing, while continuing to increase human resources across the country.

### Study limitations

A small sample size means data saturation may not have been met, but most stakeholders directly involved in decisions around hepatitis B policy in Vanuatu were interviewed. Whilst it is likely that sufficient people were interviewed to gain a holistic understanding of issues related to midwife or nurse-delivered interventions care must be taken not to assume that our data represents all stakeholders’ perspectives on this model beyond these settings. Frontline nurses and midwives who would be implementors of any changes and stakeholders from provinces without PPBY intervention sites were not interviewed and additional research is required to understand implementation of this task-shifting in other provinces, as well as among midwives and nurses implementing this care, and pregnant women living with hepatitis B. This work is planned.

‘Internal reliability’ – understood as whether the interpretations and meaning drawn from data collected are true reflections of the experiences and meanings of people interviewed [48] – is an important concern in rigorous qualitative research. This was enhanced in several ways. Interviewers have previously conducted qualitative research in Vanuatu and were trained and supported to conduct this research. Interviews were conducted in English or Bislama, by interviewers with whom participants had prior working relationships, which facilitated trust, rapport and open communication. Interviewers and translators were involved in data analysis, which enabled deeper interpretation of data.

### Implications for policy and practice

Our findings identify several opportunities to enhance task shifting to support PMTCT of hepatitis B in Vanuatu and other similar settings. First, improved education, training and ongoing support for nurses and midwives would support effective task-shifting. One approach highlighted by several participants included trainers visiting health facilities to adapt strategies to the specific and unique setting of each health facility, instead of always bringing people to a central location. Second, roll out of health information, education and communication activities among pregnant women, delivered in a way that is culturally appropriate and ensures ongoing minimisation of stigma, would enhance community understanding of the availability of integrated services and the importance of accessing them. Third, scale up of task-shifting could be supported by implementation research to ensure ongoing quality improvement, effective adaptation to different provinces and socio-cultural settings, and support roll out to new settings across the Pacific where evidence is also lacking. Fourth, any scaling of midwife- or nurse-led hepatitis B interventions would require comprehensive associated policies, including around licensing for prescribing and formal task-shifting arrangements for compensation.

## Conclusions

The results of this study are encouraging, with strong indications that a midwife- or nurse-led approach for PMTCT of hepatitis B is both acceptable and feasible. Solutions to identified challenges inhibiting long-term sustainability of this model need to be found to ensure continuous access to high-quality health care, particularly in relation to under-staffed and under-resourced facilities, and supply chain issues. Applied prospectively through PRISM and RE-AIM, this study demonstrates how implementation science frameworks can identify system-level preconditions for task-shifting before scale-up, offering a replicable approach for similar settings across the Pacific and beyond.

## Supporting information

S1 File COREQ checklist. (PDF)

S2 File Topic guide. (PDF)

S1 Checklist Inclusivity checklist. (DOCX)
