# Supplementary material for: Stakeholder perspectives on task shifting to prevent mother-to-child transmission of hepatitis B: A qualitative exploration of midwife- and nurse-led interventions in Vanuatu
Source: PLOS Glob Public Health. 2026 Jul 8;6(7):e0006450. doi: 10.1371/journal.pgph.0006450 (PMC13345262; doi:10.1371/journal.pgph.0006450)
Supplement: S2 File — (PDF) [file pgph.0006450.s002.pdf]

Understanding acceptability of midwife-delivered care for hepatitis B prophylaxis during pregnancy in Vanuatu: A qualitative study

Interview guide for health service and system stakeholders

Aims and objectives

Overall aim: to examine feasibility and acceptability of midwife-delivered provision of hepatitis B prophylaxis treatment during pregnancy to prevent mother-to-child hep B transmission in Vanuatu.

Specific objectives of this interview are to gather the perspectives of clinic managers, policy makers and other national stakeholders on factors relating to (a) clinics, (b) personnel, (c) health system and (d) communities on feasible and sustainable delivery of the model of care

Materials and supplies needed

- Consent forms (one copy for participant, one copy for the team)
- Question guide for interviewer
- One digital recording device with batteries
- Spare battery for recording device
- Notebook for note-taking and pens
- Refreshments and incentives

Structure and timing

**Section**	**Aims**	**Timing**
S1: Warm up	Build rapport; make interviewee comfortable talking; check informed consent; understand the professional roles and experiences of interviewees	5 mins
S2: Hep B diagnosis and treatment in pregnancy	Understand standard current practice on management of Hep B in pregnancy	10 mins
S3: Exploring midwife-led model	Examine clinic managers' and other stakeholders' perspectives on the midwife-delivered model of care for Hep B prophylaxis during pregnancy:<br>**unpack acceptability - how feel about the intervention, perceived effort to participate, alignment of model with women's value system, understanding of intervention and how it works, opportunity costs (what give up to deliver role), likely to achieve purpose, confidence to fulfil model requirements*<br>**unpack feasibility through REAIM (reach within the target population; effectiveness or unintended consequences; adoption by staff/settings/systems/communities; maintenance over time) and PRISM (multi-level perspectives; characteristics of recipients; Implementation and sustainability infrastructure)*	40 mins
S4: Cool down	Aim to leave stakeholders in same mental/emotional state as started interview; check any other questions; any other points want explaining	5 mins

RUN THROUGH PARTICIPANT INFORMATION SHEET AND CONSENT FORM
GAIN INFORMED CONSENT
Remind the participant that this interview will be recorded and transcribed.

S1: Warm up

Can you tell me a bit about your current role?
Can you tell me a bit about the organisation where you work?

S2: Hep B diagnosis and treatment in pregnancy

How important is it to support pregnant women with hep B treatment during pregnancy? Why?
Can you talk us through how Hep B in pregnancy is typically managed in your clinic / Vanuatu?

S3: Acceptability, feasibility and impact of the midwife-led model

Explain that we are now going to dive into the midwife-led model of care in more detail. We're keen to understand your thoughts on the model of care and any issues affecting acceptability, feasibility and sustainability of the model. Interviewer to describe the model for women who test positive for Hep B:

- Therapeutic: Tenofovir disoproxil fumarate (TDF); only antiviral therapy for hep B infection available in Vanuatu.
- Oral administration: 300 mg table to be ingested once daily
- Duration treatment: From the second trimester of pregnancy (somewhere between weeks 12-28) until completion of the infant HBV vaccination series (14 weeks after delivery); total weeks ranging from 26-42
- Prescription: Dispensing 5 weeks at a time (35 tablets)
- Engagement with midwife: Monthly, in line with ANC recommendations, with preferred approach for dispensing is tied to routine ANC or post-natal appointments and childhood vaccination.
- Location of dispensing: Women's closest ANC, provided through health clinics. This may be walkable or may require a bus (tied to scheduled ANC visits so shouldn't require additional out of pocket expenses). The MOH is designating "PPTCT sites" with around 10 per province which will be targeted for training and program implementation. These are the larger health centres.

What are your initial thoughts on clinics/health systems being able to deliver this model of care to pregnant women with Hep B in Vanuatu? Why (not)?

To what extent do you think midwives will be able to deliver this model of care? Why (not)?

Do you think this model of care can be delivered in hospital and community-based ANC settings? Where? Why?

To what extent do you think that pregnant women with Hep B will be able do the following things:
*Attend routine ANC meetings, post-natal appointments, childhood vaccinations and receive prescription?
*Taking daily tablets orally?

Please can you describe any barriers, challenges or concerns you have regarding the ability of clinics/health systems to adopt / deliver this model of care as routine practice. Service barriers? Practitioner based barriers? Client-based barriers? Health system-based barriers? External factors?

To what extent do you think this model of care can be implemented across Vanuatu? Why (not)?

Do you think this model of care will reach all pregnant women with Hep B? Why (not)?

What type of clinical mentoring, referral or other processes might need to be established for helping midwives to manage complex cases?

S5: Cool down

Any suggestions about what could be changed to improve this model of care?

Any questions for us? Any information needed? Anything you wanted to talk about that we've not discussed?

Thanks and close
